# Improving the quality and use of immunization and surveillance data: Summary report of the Working Group of the Strategic Advisory Group of Experts on Immunization

**DOI:** 10.1016/j.vaccine.2020.09.017

**Published:** 2020-10-27

**Authors:** Heather M. Scobie, Michael Edelstein, Edward Nicol, Ana Morice, Nargis Rahimi, Noni E. MacDonald, M. Carolina Danovaro-Holliday, Jaleela Jawad, the SAGE Working Group on Immunization and Surveillance Data Quality and Use

**Affiliations:** a Centers for Disease Control and Prevention, Atlanta, USA; b Public Health England (PHE), London, United Kingdom; c Burden of Disease Research Unit, South African Medical Research Council, Cape Town, South Africa; d Health System and Public Health Division, Faculty of Medicine and Health Sciences, Stellenbosch University, South Africa; e Independent Consultant, San Jose, Costa Rica; f Shifo Foundation, Sweden; g Dalhousie University, IWK Health Centre, Canada; h World Health Organization, Geneva, Switzerland; i Public Health Directorate, Ministry of Health, Bahrain

**Keywords:** Immunization, Vaccination, Vaccine-preventable disease, Vaccination coverage, Surveillance, Data quality, Data use, Information systems

## Abstract

Concerns about the quality and use of immunization and vaccine-preventable disease (VPD) surveillance data have been highlighted on the global agenda for over two decades. In August 2017, the Strategic Advisory Group of Experts (SAGE) established a Working Group (WG) on the Quality and Use of Global Immunization and Surveillance Data to review the current status and evidence to make recommendations, which were presented to SAGE in October 2019.

The WG synthesized evidence from landscape analyses, literature reviews, country case-studies, a data triangulation analysis, as well as surveys of experts. Data quality (DQ) was defined as data that are accurate, precise, relevant, complete, and timely enough for the intended purpose (fit-for-purpose), and data use as the degree to which data are actually used for defined purposes, e.g., immunization programme management, performance monitoring, decision-making. The WG outlined roles and responsibilities for immunization and surveillance DQ and use by programme level.

The WG found that while DQ is dependent on quality data collection at health facilities, many interventions have targeted national and subnational levels, or have focused on new technologies, rather than the people and enabling environments required for functional information systems. The WG concluded that sustainable improvements in immunization and surveillance DQ and use will require efforts across the health system — governance, people, tools, and processes, including use of data for continuous quality improvement (CQI) — and that the approaches need to be context-specific, country-owned and driven from the frontline up. At the country level, major efforts are needed to: (1) embed monitoring DQ and use alongside monitoring of immunization and surveillance performance, (2) increase workforce capacity and capability for DQ and use, starting at the facility level, (3) improve the accuracy of immunization programme targets (denominators), (4) enhance use of existing data for tailored programme action (e.g., immunization programme planning, management and policy-change), (5) adopt a data-driven CQI approach as part of health system strengthening, (6) strengthen governance around piloting and implementation of new information and communication technology tools, and (7) improve data sharing and knowledge management across areas and organizations for improved transparency and efficiency.

Global and regional partners are requested to support countries in adopting relevant recommendations for their setting and to continue strengthening the reporting and monitoring of immunization and VPD surveillance data through processes periodic needs assessment and revision processes. This summary of the WG’s findings and recommendations can support “data-guided” implementation of the new Immunization Agenda 2030.

## Highlights

• Reported to SAGE on immunization and surveillance data quality & use, October 2019.
• Improvements since 2011, but gaps persist in data quality (fit-for-purpose) and use.
• Improving data quality and use requires better governance, people, tools, and processes.
• Better use of existing data needed to continuously improve programme performance.
• Report relevant for “data-guided” implementation of the Immunization Agenda 2030.

## 1 Background

Concerns about the quality and use of immunization and vaccine-preventable disease (VPD) surveillance data for decision-making have been highlighted on the global agenda for more than two decades [1], [2]. In 1998, following the identification of inconsistencies in national-level vaccine coverage data reported to the World Health Organization (WHO), the Strategic Advisory Group of Experts on Immunization (SAGE) recommended intensified efforts to improve immunization data quality, leading to the conception of WHO/UNICEF estimates of national vaccine coverage (WUENIC) [3], among other efforts.

Countries need quality data for immunization programme management and decision-making and to meet the ambitious regional and global vaccine coverage and disease elimination goals, such as those that were outlined in the Global Vaccine Action Plan (GVAP) [4]. Subsequently, the GVAP companion document “Global Routine Immunization Strategies and Practices (GRISP)” was developed to “reassert routine immunization as the foundation for sustained decreases in morbidity and mortality from vaccine-preventable diseases across the life-cycle of all individuals” [5]. GRISP emphasized the importance of improving data quality and use to guide programme management and improvement.

Looking forward, the emphasis on data quality and use will become even stronger with the development of the Immunization Agenda 2030 (IA2030), which was endorsed by the World Health Assembly in August 2020 and sets a global immunization strategy to achieve a “world in which everyone, everywhere, at all ages, fully benefits from vaccines for their health and well-being” and which highlights “data-guided” as one of its four core principles [6]. Improved information systems and quality data will also be critical to measuring progress towards achieving the Sustainable Development Goals (SDGs) and Universal Health Coverage (UHC), such as improvements in Primary Health Care across the life-course and equity of service delivery [7], [8], [9], [10]. Sustainable improvements will require a whole-systems approach that includes people, tools, governance, and processes including for continuous quality improvement [11].

As a concrete measure towards improving data quality and use, SAGE established a Working Group (WG) on the Quality and Use of Global Immunization and Surveillance Data in August 2017 tasked with (i) taking stock of immunization and VPD surveillance data availability, quality, and use at country, regional, and global level; (ii) assessing unmet monitoring needs at the global and regional level; (iii) assessing any gaps in the standards and guidance on immunization monitoring and surveillance; (iv) reviewing evidence on the factors limiting data quality and use as well as the effectiveness of related interventions; (v) reviewing the status of information systems and modern technology to support the collection, management, analysis, and use of immunization and surveillance data; and (vi) identifying any gaps in evidence and creating a research agenda [12]. The WG’s final findings and recommendations were presented to SAGE in October 2019 [13]. This article aims to summarize and disseminate the recommendations to national and global stakeholders to support implementation of the IA2030.

## 2 Methods

We considered vaccination coverage (the proportion of an eligible population who is vaccinated), immunization programme process indicators (e.g., vaccination sessions), vaccine supply, and VPD surveillance data to be within the scope of the review. Vaccine safety and financial data were excluded.

We synthesized evidence across various landscape analyses, literature reviews, country case studies, surveys of immunization experts, and a data triangulation analysis to address the tasks (i-vi) outlined above. Detailed methods and reports for these reviews and analyses can be found in the full WG report (chapter 1.2) and the SAGE website [14]. In brief, the literature reviews that were synthesized included systematic reviews, as well as a “realist review” and several “scoping reviews” on key topics, including barriers limiting data quality and use, and what works to improve data use [15]. Although differing slightly in terms of methodology, all literature reviews included searches of electronic databases (e.g., Pubmed) to identify relevant published literature and included the grey literature. Where there was a paucity of high-quality evidence, the WG employed expert opinion and consensus. Key informant interviews and self-administered questionnaires were conducted among 22 immunization experts working at all levels of WHO, partner agencies, ministries of health, and experts on vaccination coverage surveys and management of humanitarian crises.

**Fig. 1:**
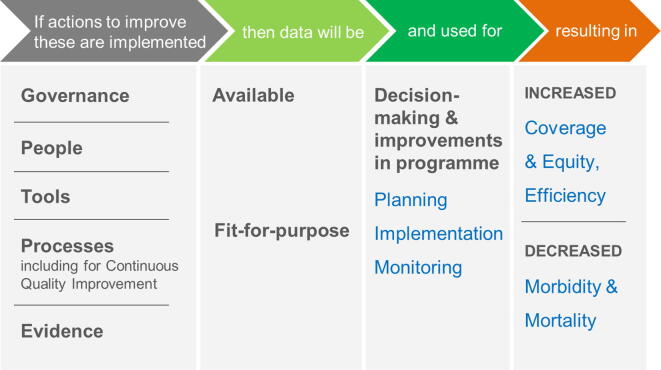
Simplified theory of change on how health system inputs lead to improvements in immunization programmes and health outcomes. Adapted from [11].

We structured our findings around a simplified theory of change (Fig. 1) adapted from the *Global Framework to Strengthen Immunization and Surveillance Data for Decision-making* [11]. The theory of change includes five health systems areas where efforts are needed — Governance, People, Tools, Processes (including for Continuous Quality Improvement), and Evidence. The recommendations made in the report aim to positively impact these areas in order to generate data that are available, fit-for-purpose and used for action, thereby resulting in increased vaccination coverage, equity and efficiency of service delivery, as well as decreased VPD morbidity and mortality (Fig. 1).

## 3 Findings and observations

### 3.1 Definitions

The WG adopted a definition of data quality as *data that are accurate, precise, relevant, complete and timely enough for the intended purpose (or “fit-for-purpose”)*, such as to monitor immunization programme performance, support efficient programme management or provide evidence for decision-making (Box 1) [16], [17]. “Data use” is the degree to which data are actually used for a defined purpose, e.g., programme management, performance monitoring, decision-making [16].

Box 1 **Attributes of immunization and surveillance data quality, as defined as “fit-for-purpose”**

**Accuracy** — Degree of agreement between a given measurement and the actual (or true) value.
• **Concurrence** (proxy) — Degree of agreement between different methods intended to measure the same construct.
• **Representativeness** (proxy) — Degree to which population measured reflects the intended target population (e.g., similar distribution of important characteristics).
• **Integrity** (proxy) — Degree to which data, once entered into the official record, are not lost, incorrectly transcribed from one record to another, or otherwise altered from the original, i.e., accuracy of stored/reported data.
**Precision** — Degree of spread among a series of measurements that is independent of accuracy
• **Consistency** (proxy) — Degree to which data attributes are free from contradiction and are coherent with other data in a specific context of use, e.g., over time for one indicator or across related indicators.
**Relevancy** — Degree to which the data collected and reported reflect what is most important to support decision-making and not in excess of what is needed so as to consume scarce resources.
**Completeness** — Degree to which all relevant data needed for decision-making are recorded and reported and therefore available for use.
**Timeliness** — Degree to which data are current and available when needed to inform decisions.
**Source***: Adapted from Bloland and MacNeil (2019) [17]*

### 3.2 Availability, quality and use of immunization and surveillance data, data-related guidance and assessment methods

There is a considerable amount and variety of immunization and surveillance-related data available nationally, regionally, and globally, though the data are not always accessible to those that need them the most [16]. At the national level, routine coverage data are available, through health management information systems (HMIS), and in some cases, stand-alone immunization reporting systems, and coverage surveys. Data for monitoring equity usually comes from periodic coverage surveys or ad hoc studies [18], [19], while data for routine monitoring of high-risk populations may not be available in most countries. Campaign coverage data are often available but may not be well-archived or in a standardized format for use. VPD surveillance data are collected nationally by integrated communicable disease surveillance systems and/or disease-specific case-based surveillance (e.g., polio, measles, rubella, neonatal tetanus). Adverse Event Following Immunization (AEFI) monitoring systems exist in some form in most countries, but may not be robust [16].

National reporting processes for administrative immunization and VPD surveillance data were considered to be functioning well, but the quality of these data is still often poor, especially in low- and middle-income countries (LMICs) [20], [21], [22], [23], [24], [25], [26]. Reporting was not always conducted according to guidelines, and the tools (forms, hardware and software) were not always available or optimal to support reporting processes [16]. Data from private providers may not be included in routine reporting [16]. The inaccuracy of denominators to calculate vaccine coverage or disease incidence rates was identified during the review as a common problem [27], [28]. In addition to concerns over VPD surveillance data quality, much of the aggregate VPD surveillance data collected lacks relevant information (e.g., age, vaccination status, laboratory confirmation) for immunization programme management [23], [26], [29], [30]. The systematic linkage of laboratory and epidemiological data for case-based VPD surveillance was identified as a current gap in many countries [31]. Finally, there is also increased demand from global partners for the collection and use of subnational and individual-level immunization and VPD surveillance data to support achieving programme objectives [32], [33].

Since 1988, the WHO-UNICEF Joint Reporting Form (JRF) has collected standardized immunization, surveillance and other programme data from all countries on an annual basis [34]. The JRF reporting and validation process is revised every other year and the data included have become more comprehensive, expanding beyond coverage and surveillance to meet new monitoring needs as the Expanded Programme on Immunization (EPI) evolves. Subnational vaccination coverage data for several vaccine-doses has been collected globally through the JRF since 2017, with known limitations [32]. Plans to switch to an online reporting system (eJRF) are ongoing and are related to the the development of WHO Immunization Information SystEm (WIISE), an integrated platform for management and visualization of coverage, surveillance and other data at the global level that is projected to improve data availability and usefulness [35]. At the global level, immunization data collected through the JRF [34] and vaccination coverage estimated for each country by WUENIC [36] remain key sources of immunization data available openly on the WHO and UNICEF websites [37], [38].

The WG identified a large number of global and regional guidance documents and standards that have been developed to address issues related to EPI monitoring, data quality and use [16], [39]. However, people working in immunization and VPD surveillance were not always aware or able to find and access relevant documents. In addition, the WG identified a number of gaps in guidance relating to various aspects of the monitoring and evaluation of vaccination programmes, including how to improve programme targets (denominators), track mobile populations, monitor vaccination across the life-course, and routinely monitor coverage equity [16].

A number of tools for countries to assess immunization data quality exist, such as the Data Quality Self-assessment (DQS) and Data Quality Review (DQR) toolkit [40], [41]. These tools have evolved in a way that has improved country ownership and interest in making data improvements, with some evidence of positive impact on data quality and use as well (Table 1) [16], [42]. A review completed by the WG identified many data quality measures by different quality attributes for immunization coverage, denominators, and surveillance data (available as an annex to the full WG report) [16]. More work is needed to define a common lexicon of definitions around data and a standard set of indicators to measure data quality and use, as part of comprehensive programme monitoring [17].

**Table 1:** History of immunization data quality assessment guidance with strengths and limitations.

**Data quality assessment guidance, year**	**Description**	**Strengths**	**Limitations**
Data Quality Audit (DQA), 2003 [124]	First immunization data quality assessment tool used by partners to validate number of children vaccinated for performance-based financing	• Quantitative measure of reporting accuracy (verification factor, VF) • Quality of the system index (QI) from assessment of data system at each reporting level • Guidance on practical recommendations for data recording and reporting	• Not a country-owned or country-led process • Small sample sizes at the district level can create large variation in the reporting verification factors • No direct observation of recording and reporting practices at health facility level
Data Quality Self-Assessment (DQS), 2005 [41]	Adaptation of DQA to assist countries to self-diagnose data quality problems at the national, provincial, or district levels in order to improve their monitoring systems	• Flexible including data review and self-designed questionnaire to assess system quality issues (including direct-observation at facilities) • Used widely and regularly by countries and is encouraged as part of EPI reviews	• Because it is adapted by countries and site selection may be biased, results are not comparable across countries • Regular widespread implementation of DQS takes effort and may not result in interventions to improve data quality
Assessing and Improving the Accuracy of Target Population Estimates for Immunization Coverage, 2015 (draft) [125]	Working draft of a guide to facilitate national immunization programmes to assess their target population estimates for vaccination coverage	• Emphasizes importance of collaboration with local statistics office • Includes assessing internal and external consistency (comparison with alternative sources, examining population growth rates and IMR)	• Low awareness of tool among key informants, and extent of use unclear • Needs to be finalized • Needs updating with practical case studies, geospatial estimates, advice about migrants
Data Quality Report Card (DQRC), 2015 [126]	Integrated data quality review tool including immunization and other programme measures (antenatal care, deliveries, population estimates)	• Annual data quality desk review for health facility level including reporting completeness, internal consistency of reported data, and external consistency of population data and coverage rates • Excel tool produced report card as output	• Prescriptive process relying heavily on Excel tool • Limited current use
Data Quality Review (DQR), 2018 [40]	Toolkit based on DQRC to assess data quality at the health facility level with unified approach to data quality across many disease control programs (TB, malaria, HIV and EPI)	• Integrated health systems approach • Encourages routine reviews of data quality built into validation checks, annual independent assessments, and periodic in-depth reviews of data quality for specific programmes • Systems assessment and module to validate data integrity in the field also included	• May be a “tick-box” exercise to satisfy those at the international level demanding attention to data quality • No programme is covered in depth (several indicators each) • Unclear basis for benchmarks of data quality analyses
Tools for monitoring the coverage of integrated public health interventions, 2017 [127]	Integrated methods and tools for monitoring coverage and data quality of immunization and deworming interventions at the local, district/municipality and national levels, published by PAHO	• Practical approach, relevant for other regions • Encourages in-depth evaluation of data quality every 3–5 years, plus annual assessments and data congruence exercises during supervisor visits • Focus on data accuracy, timeliness and completeness, and systems assessment	• Long document with many modules – can be difficult to navigate
Health facility analysis guidance for immunization programme managers, 2018 (draft) [128]	Practical analysis guidance on performance monitoring and data quality related to DQR, but specific to immunization	• Relevant for routine monitoring at the national and subnational level • Accompanying module for DHIS2	• Mostly implemented in African Region • Needs to be finalized
Handbook on the use, collection, and improvement of immunization data, 2019 (draft) [54]	Comprehensive immunization monitoring handbook building on the DQR and including a number of other immunization-specific topics for national level	• More detailed and is less prescriptive than DQR • Includes root-cause analysis to tailor recommendations and feed into a data improvement plan	• Broad, so topics are not covered in depth • Needs to be finalized
PRISM: Performance of Routine Information System Management, 2019 [123]	Toolkit revised from 2011 version to assess routine health information systems, data quality and use, including indicators from reproductive health TB, malaria, HIV and EPI	• Integrated periodic health information systems assessment toolkit including reporting & data completeness, accuracy of facility reports • Also assesses data management, analysis & use • Systems assessment addresses technical, organization and behavioral determinants	• Periodic assessment approach

Abbreviations: DQA = Data Quality Assessment, DQS = Data Quality Self-Assessment, DQRC = Data Quality Report Card, DQR = Data Quality Review, DHIS2 = District Health Information System 2, EPI = Expanded Programme on Immunization, HIV = Human Immunodeficiency Virus, IMR = implied mortality rates, TB = tuberculosis, PAHO = Pan American Health Organization.

### 3.3 Factors limiting and the effectiveness of interventions to improve access, quality and use of immunization and surveillance data

Data quality loss or failure to share and use data can occur through multiple mechanisms at all levels of the health system. Data quality loss can result from failure to record properly, transcription or calculation errors, missing or outdated forms, procedural gaps (e.g., not including private sector), lost or damaged records, inaccurate denominator data, and intentional falsification [17], [43]. Root causes associated with poor data quality include gaps in health worker capability and motivation, performance-based targets, unsupportive leadership, lacking a culture of data use, poor information system design, overly complex tools, inadequate policies and resources, and suboptimal processes for data collection and reporting, including supervision and feedback [17], [29], [30], [43], [44], [45].

Types of barriers to sharing data locally and internationally include technical (inadequate interoperability, standards, archiving procedures); motivational (lack of incentives, trust between data providers and users, or resources/time needed); economic (e.g., potential negative economic effects); political (bureaucratic hurdles, lack of political will); legal; and ethical barriers [46], [47].

Failure to use data can result from a lack of any of the following: confidence in the quality of available data, data analysis and interpretation skills, understanding on how to use data to monitor and improve immunization programmes, or a culture of information use (“data use culture”) for various reasons [17], [43], [44], [48].

### 3.4 Emerging issues and recent successes related to improving immunization and surveillance data quality and use

There are many emerging issues for data quality, including the need for more accurate population estimates to ensure coverage accuracy especially as coverage increases [49] and to include private-sector data in reports [50]. Access and full use of VPD surveillance data that is relevant to immunization programme planning and decision-making remains limited [23], [26], [33]. Another monitoring challenge for immunization programmes is moving from infant vaccination towards a life-course vaccination approach [51]. As global vaccination policy moves to promote equity across subpopulations and geographic areas, appropriate indicators and routine monitoring beyond vaccination coverage need to be developed and implemented [52]. The development of global and regional guidance on monitoring immunization inequalities using survey data is a step in the right direction [19]. More accurate target population estimates that include migrant and marginalized populations, also remain a pressing need at the operational programme level [7], [52].

At the global level, developments noted by the WG that could potentially help improve the quality and use of immunization and surveillance data include new electronic platforms, like WIISE, new guidance like on Electronic Immunization Registries [53], DQR [40], WHO Immunization Data Handbook [54] and related bi-lingual (English and French) distance-based learning initiatives using the “WHO Scholar” platform, consisting of short video lectures, discussion sessions, and real-life projects supported by peer-learning and mentoring for enrolled students mostly working at the national, subnational, and health facility levels [55], [56]. The revised VPD Surveillance Standards [33] and draft global Comprehensive VPD Surveillance Strategy [57], a companion document to the IA2030, also emphasizes system-design based on the data required to achieve surveillance objectives and greater access and use of information to manage immunization programmes.

At the national level, many countries are adopting online electronic health information systems resulting in improved data management and access, and some countries are demonstrating success with electronic immunization registries (and others less so) [42], [53], [58], [59], [60], [61]. Large countries like India and China have demonstrated success at using triangulation of immunization and surveillance data to identify immunity gaps [16], [62]. Denominator improvement projects have occurred in many countries, including use of geospatially modeled estimates [63], [64], [65], [66], [67]. While there is increased uptake of Civil Registration and Vital Statistics (CRVS) in some countries, progress in LMICs is likely to be slow [68], [69], [70], [71], [72].

### 3.5 Working group perspective on the current needs for improving data quality and use

A recent realist review found that multicomponent interventions are most effective for improving health data quality and use [42], similar to the findings of a systematic review of interventions for improving health worker performance [73]. For example, no impact has been observed from technological interventions alone, without the related capacity building [42]. Further, employing a health system approach that addressed multiple areas (e.g., standards, hiring data managers, data review meetings, and supportive supervision) was found to be more likely to succeed and be sustained over long-term [42], [73].

The perspective of the WG is that sustainable improvements in data quality and use require effort across the healthcare system (i.e., governance, people, processes, tools), not just new technological solutions. Continued exclusive focus on *low-hanging fruit* will not address the root issues and achieve sustainable change. For this reason, it is relevant to consider multicomponent interventions within and across the five key areas of Governance, People, Tools, Processes for Continuous Quality Improvement, and Evidence towards improving data quality, access and use as part of a health systems approach. The approaches need to be context-specific, country-owned and driven from the frontline up.

## 4 Governance: Strengthening governance of data collection, access, and use

Within the area of governance [74], there are several fundamental factors that enable collection of high-quality data, as well as access and use (Box 2). Having strong policies (e.g., for eHealth) and accountability mechanisms in place that govern all key aspects of data collection, access, integration, and use is important to develop immunization and VPD surveillance information systems that produce high-quality, credible data that are useful to monitor and improve programmes [11], [75], [76]. National standards governing all stages of data generation, use and sharing (both within organizations in country and internationally) that consider privacy and confidentiality are needed [46], [47].

Box 2 **Enabling factors for governance of immunization and surveillance data systems.**

**Leadership & political will** to establish processes for reporting & data quality improvement
**Accountability** for clearly defined terms of reference & deliverables, mechanisms for monitoring
**Standards & user-friendly guidance** for tools & processes, including feedback
**Coordination** structures or mechanisms to facilitate efficient communication & work across units
**Sharing/access of data and information** to those who need it for planning and decision-making
**Resources** allocated to support all aspects of data collection/management

Improving data quality and use requires leadership and commitment from national governments, such as sufficient resources and supportive policies and regulations, to facilitate a “data use culture” for continuous quality improvement, as well as a willingness to improve data quality — even if it initially leads to lower reported performance [77]. Coordination and collaboration between different units dealing with immunization and surveillance data (e.g., immunization programme, surveillance units, and laboratories in the public, private and not-for-profit sectors) is crucial to establish efficient, sustainable information systems that avoid data fragmentation and duplication [46], [78]. The full WG report (chapter 3) includes several examples of the positive impact of robust national governance processes on vaccination data quality and use [16].

## 5 People: Building capacity and capability of the health workforce in data collection and use

Issues around health workers’ competencies related to data management have been widely documented, including the lack of sufficient capacity, capability and motivation in data collection, analysis, interpretation and use [24], [43], [79], [80], [81]. These issues are key factors limiting the quality and use of immunization and VPD surveillance data [29], [30], [44], [82], [83]. Data quality at all levels ultimately depends on the quality of data collection at the health facility level, and thus data quality interventions, including workforce planning and capacity-building must specifically target the local level [17].

Frontline staff are often over-burdened with multiple responsibilities, including data collection for vertical programmes [14]. They spend a third of their time on data-related activities such as recording and reporting [84], [85], which often compete with clinical duties, thus impacting the quality, completeness and timeliness of reporting [86], [87]. The data demands of global partners are not always aligned with the priorities of national immunization programmes and can further burden frontline healthcare workers [14], [30], [88]. The focus on technology — rather than on the people who drive information systems — has often led to the development and implementation of complex health management information systems, or tools, without sufficient attention to human resources required to run them [30], [43], [89].

Improving this situation requires a multi-pronged approach — including improved workforce planning, pre-service and in-service training, with regular reinforcement through supportive supervision and effective feedback [73], [89], [90], [91]. Adequate resourcing and dedicated person-time for data-related tasks also need to be taken into consideration [92], [93]. A global framework of immunization workforce competencies has been developed that may be helpful for this purpose [93], [94]. Some countries such as India [62] and Botswana [95] have dealt with the issue by creating a cadre of health information personnel specifically trained and dedicated to managing and analyzing data; more evaluation of the usefulness and sustainability of this strategy is needed.

There are several recommended curricula available world-wide for pre-service training of health professionals that include modules on the collection, analysis, management, and use of immunization data. These include the “EPI Prototype Curricula for doctors and nursing/midwifery schools” in the WHO African Region [96], [97] and the Mid-Level Management Course for EPI managers [98], which has been recommended for use both for pre-service training and for certifying professionals for practice [99].

However, findings from a scoping review on pre- and in-service training on immunization data management found that current pre-service training programmes often do not adequately prepare health workers to carry out data-related tasks, nor has most in-service training on data had any major impact in improving the skills and practices of health workers [81]. Governments therefore need to make a dedicated effort to provide effective and continuous competency-based training on the generation and use of health data [92], [93], incorporating adult learning theory and based on the data-related responsibilities required at different levels of the health system. Global partners should think strategically about the added value of additional data requests, and prioritize accordingly.

**Fig. 2:**
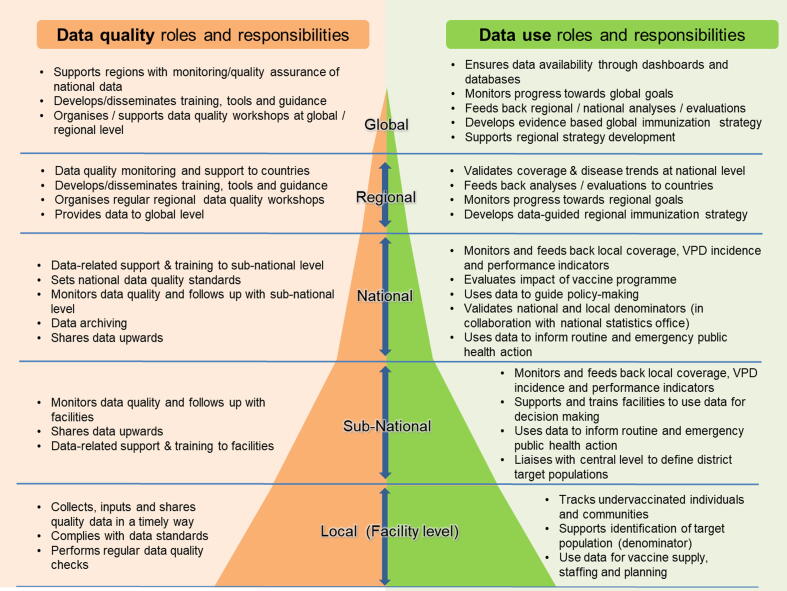
Data quality and use roles and responsibilities by level of immunization & surveillance program. This schematic was developed by the SAGE Data Working Group.

The WG developed a framework that defines the roles and responsibilities of health workers in collecting, analyzing and using immunization data from the facility to the global level in order to assist countries in planning their capacity-building activities related to immunization data and information systems (Fig. 2). Interventions to address issues around data quality and insufficient skills sets, including plans to hire new staff, should be focused on elements of these competency frameworks [93], [94].

## 6 Tools: Aligning information systems and technological innovations with local context & programme needs

Advances in information and communication technology (ICT) have led to a multitude of tools developed to address deficiencies in data quality, availability and use (Table 2), which are described in more detail in the full WG Report (chapter 5) [14]. Some of these tools, in particular information systems and decision-support tools (e.g., dashboards), can improve the quality and use of data [42] and are in use in many countries globally, including in LMICs [100]. However, many tools do not go beyond the pilot stage, thereby wasting financial and human resources [101]. The reasons include over-enthusiasm for adopting technological innovations without defining the problem to address, a lack of rigorous evaluation, as well as insufficient consideration of critical “readiness” factors that are pre-requisites for successful implementation [53], [59]. Readiness factors include the governance structures and procedures needed to support the new technology, the human resource needs to operate and use it, integration with existing systems, infrastructure requirements, and financial sustainability.

**Table 2:** Examples of using modern technologies for immunization and VPD surveillance with strengths and limitations.

**Category**	**Examples**	**Strengths**	**Limitations/further evaluation needed**
Immunization Information Systems	• Electronic immunization registries • Health management information systems	• Can improve data quality and use • Helps improve vaccination schedule completion and timeliness through clinical decision support tools • Can improve access and equity by allowing coverage monitoring at local or individual level • Can support AEFI case investigations and causality assessments; vaccine effectiveness studies	• Impact varies based on country infrastructure and readiness
Digitization of paper records	• Scanning facility paper forms to generate child registries and monthly reports (e.g., Smart Paper Technology) • Capturing images of paper forms on mobile devices	• User-friendly • Time saving overall (i.e., data entry) • Availability of digital archive for data cleaning	• Independent evaluation needed
Decision-support tools	• Dashboards	• Can improve data availability, quality and use • Helps monitor and triangulate performance, data quality, logistics data	• Can be expensive • Needs to be accompanied by training for impact
Logistic management information systems	• Digital supply chain	• Helps reduce duration of stockouts • Results in better stock management • Lessens errors	• Impact varies based on country infrastructure, readiness, and disaggregation of data
mHealth	• Electronic birth and vaccination registries • Automated reminders, defaulter tracking • SMS-based reporting of AEFIs • Data collection for vaccination campaigns and community-based surveillance • Data feedback to frontline healthcare workers	• Can improve defaulter tracking and timely vaccination • User-friendly • Potential to directly reach caregivers	• Limited evidence for immunization, except for SMS reminders • Willingness of caregivers to receive SMS or other types of automated reminders varies by setting
Media based	• Weekly videos to healthcare workers reminding what data to collect (and how)	• Improves sensitivity of surveillance • Improves reporting completeness	• Expensive to scale-up
Geospatial	• Estimating demographic data for microplanning • Tracking seasonal population variations (e.g., through call data records) • Tracking campaign vaccination to identify missed areas (also surveillance activities conducted)	• Helps improve denominators, resulting in improved quality of coverage estimates • Identification of immunization gaps through improved microplans • Better understanding of mobile populations	• Further evaluation needed to see if useful for programme planning
Predictive analytics	• Defaulter prediction • Modelled coverage estimation • Predictive outbreak detection	• Complements routine data sources for coverage • Better precision than administrative coverage • Earlier detection of VPD outbreaks	• Further development and evaluation needed to see if useful for programme planning

Abbreviations: AEFI = adverse effects following immunization, mHealth = mobile health, SMS = short message service, VPD = vaccine preventable disease.

In general, there is a trend towards collecting, processing, and analyzing immunization and VPD surveillance data as part of integrated systems, rather than stand-alone systems developed specifically for vaccine programmes [16]. An integrated approach creates efficiencies from similar data requirements across health programmes, which facilitates data linkage and monitoring along the continuum of care. However, when integrated systems do not meet the needs of immunization programmes, the immunization components of integrated tools may be underutilized and parallel EPI information systems may continue, thus increasing the burden of data collection, reporting, and management [16], [102]. Effective integration of immunization data tools with other information systems requires optimal coordination across health programs and the establishment of information system standards, including minimum information datasets and interoperability frameworks, as well as protocols for data sharing and protection [102]. Decision-support tools, such as electronic dashboards for routine immunization monitoring, have shown promise and are particularly helpful when incorporated into information systems [42], [103].

New technologies can have a positive impact on the quality and use of immunization and surveillance data. However, these interventions are not magic bullets, and are unlikely to be adopted by countries in the long-term or to lead to long-lasting data improvements unless other readiness factors and conditions are in place [53], [104]. Innovative approaches are also more likely to lead to improved data use when they address a specific need, and when they are implemented as part of a package of interventions including standards, training, etc. [42].

Rigorous evaluation of these tools has not been conducted systematically, but is essential because the impact of new tools is not always obvious [53], [104]. Where evaluations have been done, they have shown, for example, that mobile-based reporting does not always improve timeliness, or that the implementation of a health management information system does not systematically lead to improved data use [42]. In general, more evidence is needed on the impact, cost-effectiveness and sustainability of interventions such as novel ICT approaches, including documented examples of decisions on when and how to scale interventions.

## 7 Processes: Using immunization and surveillance data for continuous quality improvement

Periodic data quality assessments (e.g., every 3–5 years) alone have had limited impact on the quality of immunization and VPD surveillance data (Table 1) [20], [42]. Newer approaches that incorporate a systems assessment (governance, people, tools, processes) and result in a data improvement plan (Table 1) [40], [54] have potential for greater impact in terms of addressing the root causes of poor data quality [43]. In addition, the WG suggests shifting from periodic assessments to routine monitoring of data quality, including automated data validation checks and analyses on electronic information systems would have greater impact in terms of improving data quality (i.e., as part of a feedback cycle including real-time data correction) [40]. Monitoring data quality indicators as part of comprehensive monitoring framework would help reinforce the importance of improving data quality, in addition to achieving coverage targets.

Some evidence indicates that data quality improvements can be driven by increased data use [40], but the WG observed that much of the data collected is underutilized. Data triangulation is “an approach for critical synthesis of existing data from two or more data sources to address relevant questions for programme planning and decision-making” [105]. We suggest that triangulation of existing data, such as surveillance, rapid coverage monitoring, and vaccine supply data should be routinely conducted for public health analysis in order to create a more granular picture of programme performance at subnational and national level [16], [106]. To this end, draft guidance has already been developed, piloted in countries, and used as part of a WHO Scholar course focused on data triangulation [107].

We propose using data for CQI of immunization programmes (not just data quality) should be the vision for the next decade, including institutionalized and sustainable mechanisms for process evaluation, supervision, and monitoring at all levels of the health system [16]. Evidence from use of CQI in healthcare outside of immunization indicates that using this approach has resulted in long-lasting improvements to use of data for decision-making, as well as improved clinical outcomes and patient satisfaction [108], [109]. Within EPI, the U.S. programme called IQIP (Immunization Quality Improvement for Providers) and an earlier iteration called AFIX (Assessment, Feedback, Incentives, and eXchange) are well-documented examples of a CQI framework [110], [111]. Examples of CQI guidance and projects in developing country settings also exist, but better documentation of the effectiveness of these approaches in different contexts and appropriate scale-up is needed [16], [108], [112], [113], [114], [115]. In general, greater emphasis on strategies to improve immunization performance alongside measuring relative improvements (e.g., coverage improvement since previous period, change in number of unvaccinated children) [16], [103], [116], [117], rather than achieving absolute targets may be helpful.

When trying to address improvements over different health system areas, the use of “maturity grids” to assess country capacities has been a helpful development in terms of prioritizing and coordinating technical support for improvement, such as those proposed for immunization programmes and VPD surveillance in Africa [118], [119]. As part of the IA2030, increasing data-related capacities to facilitate data-guided decision-making will be critical for achieving goals on equity and vaccination across the life-course so that every person benefits from vaccination and no one is left behind [6].

## 8 Evidence: Filling gaps in evidence around data quality and use

Several gaps in evidence and knowledge concerning key aspects affecting the quality and use of immunization and VPD surveillance data were identified. Important challenges encountered by the WG included the lack of a framework for what data is needed for immunization programmes at different levels, the lack of consensus on the definition of “data quality,” and the lack of monitoring indicators related to “data use.” To address these fundamental gaps, the WG has proposed a working definition and outlined attributes of data quality and associated indicators, as well as uses of data by level in order to advance the discussion. Further field-testing and feedback from users are needed before key indicators can be adopted as part of any global monitoring framework, e.g., for IA2030. Of note, relevant data quality indicators are likely to differ by context and level.

Another fundamental challenge is the lack of evidence on how better data quality and use leads to better decision-making and better immunization programme performance, though examples exist from other fields [108], [109], [120], [121]. The IDEA project created an evidence gap map that highlighted greater existing evidence for the impact of interventions on improved immunization data quality and availability, but less existing evidence on what works to support immunization data-guided decision-making, particularly at the facility level [42].

The WG noted that much of the evidence reviewed regarding interventions designed to increase data quality and use generally lacked robust evaluations. There was very limited evidence on the effectiveness, cost-effectiveness and sustainability of interventions which aim to improve data quality and use. For example, despite the many pilots of novel ICT approaches for data collection, processing and reporting, few documented examples exist of evidence-based decisions on when and how to scale interventions. Some of the better examples of evaluating and scaling of interventions have been documented through the BID Initiative and the BID Learning Network [122].

A research agenda was developed by the WG and is outlined in Table 3 by each of the key areas of governance, people, tools and processes including CQI. Research needed to fill gaps on specific issues, such as processes for improving and incorporating local immunization targets (denominators), the use of triangulation and geospatial approaches for immunization monitoring and planning, and the role of serosurveys as part of immunization programme monitoring were also highlighted. More detail is provided in the full WG Report of the SAGE WG on the Quality and Use of Immunization and Surveillance Data (chapter 7) [14].

**Table 3:** Research agenda for immunization data quality and use.

**Key areas**	**Priority research questions**
Governance	• Which data are most useful at different levels in different contexts? • What are the technical and non-technical barriers to accurate denominators and numerators and how can they be overcome? • How can special populations (e.g., migrants, asylum seekers) be enumerated and monitored for vaccination (lessons from polio, NGOs)? • What are the best practices for estimating target populations and monitoring coverage for age groups beyond infancy? • What are the factors for success/failure of systematic efforts to improve data quality and use in different contexts?
People	• What is the effectiveness, cost effectiveness, and sustainability of interventions aimed at strengthening data-related workforce capacities (e.g. *Immunization Competencies Framework*)? • What are the barriers and enablers to health workers collecting high quality data and using to improve vaccination delivery? • Which incentives lead to both improved data quality and programme performance? • What are the best practices for immunization and surveillance data-related capacity-building? • What are the strengths and weaknesses of having immunization and surveillance data collected, managed and analyzed by a cadre of health information personnel vs. programme-specific staff?
Tools	• What tools are actually needed and helpful for health workers to do job in different contexts? • What is needed for integrated systems to meet the needs of immunization and VPD surveillance programs? • What is the effectiveness and cost effectiveness of technologies to improve data quality and use in different contexts? • What is the effectiveness and cost of GIS and other methods for improving population denominators? • What are the best practices and outcomes of scaling of novel technologies, including the replacement of conventional data tools?
Processes, including for continuous quality improvement (CQI)	• What is the feasibility and utility of implementing data quality and use indicators for routine monitoring at different levels? • What are the relevant data quality assessment/validation approaches for VPD surveillance data? • What is the impact of relative vs. absolute targets on program improvement and avoiding perverse incentives that inflate coverage? • What is feasibility and impact of triangulating different data, especially coverage with VPD surveillance and vaccine supply, in different contexts in terms of improving data quality and data use for programmatic decision-making? • What is the impact of data quality and use interventions incorporating quality improvement cycles or assessment/feedback approaches? • What are the most effective multi-component approaches to improving immunisation and surveillance data quality and use?
Other	• What are the best modelling approaches for WUENIC, including incorporation of other inputs, such as vaccine supply data? • What is the feasibility of validating modeled subnational coverage data and usefulness in overcoming issues with administrative data quality? • What is the feasibility of integrating vaccination coverage and VPD serosurveys with other large surveys/serosurveys (HIV, malaria)? • What are the best approaches to triangulate seroprevalence, coverage estimates, and other data? • What is the feasibility and utility of new laboratory technologies with improved performance characteristics for serosurveys (point-of-care, multiplex, capture ELISAs with improved sensitivity and specificity)? • See also the vaccination coverage survey research agenda reported elsewhere [129]

## 9 Conclusions

There is no shortage of immunization and VPD surveillance data, at all levels — local, national, regional, and global. However, data are not being used optimally to inform public health action, either because data are not accessible where needed, are of insufficient quality, or are used insufficiently in routine decision-making because of a lack of “data use culture.” Greater data use can result in better quality data and ultimately contribute to better immunization programme performance by better identifying and targeting those who are eligible for vaccination [11], [42].

Increasing and improving data use — and ultimately the performance of the immunization programme — can come about through strengthening the data-related skills and knowledge of health workers and by making better use of a diverse range of available, often-underused data, including through data triangulation. Triangulation of independent sources like coverage, vaccine stock, and surveillance data helps address and overcome the limitations of individual data sources and enables the use existing data for improved programme management and decision-making. For programme monitoring, we suggest moving beyond an exclusive focus on absolute performance targets to assessing relative improvements in programme performance and data quality at various levels.

In light of an absence of a consensus definition of immunization data quality, we suggest a pragmatic definition of “fit-for-purpose,” or good enough for performance monitoring, programme management, or decision-making. Historically, the immunization data quality debate has been focused on coverage accuracy, rather than producing data of sufficient quality to help improve vaccination and disease prevention. In order to achieve impact, we need to refocus on the underlying causes of insufficient data quality and use at different levels. Because data quality ultimately depends on the quality of data collection at the point of vaccination, data quality and use interventions must target the local level where data collection occurs.

Creating a strong “data use culture” from the local to the global level, where data is collected, reported, analyzed, used for decision-making, and fed-back for improving the delivery of an immunization programme would go a long way in driving data quality upwards. Such a data use culture emphasizes moving beyond sporadic data quality reviews and assessments (often perceived as “tick box” requirements) that treat quality data as an outcome, to supportive continuous quality improvement interventions that demonstrate the public health impact of better data quality and use to those who collect the data. Reasons for suboptimal data quality and use are context-specific and multi-faceted, so it is necessary to identify and address behavioral and organizational challenges along with building technical capacity [45], [123].

To achieve sustainable improvements in data quality and use, a health systems approach is needed. The SDGs and improving Primary Health Care to achieve UHC [9], [10] are fundamental touchpoints for many of the needed changes, including robust information systems, capacity-building on data, and use of data for continuous quality improvement. It is critical that efforts to improve immunization data quality and use be integrated with broader efforts to improve the overall performance of the immunization programme and larger health system.

In order to promote a data use culture that maximizes the utility of continuously improving data to achieve maximum population protection through robust, data-guided immunization programmes, we proposed specific recommendations under eight broad categories, many of which are for national and subnational levels (Table 4) [16]. These recommendations were endorsed by SAGE in October 2019 [13] and will support “data-guided” implementation of the IA2030, within the broader efforts of UHC and PHC [6], [9], [10]. We encourage countries, with the support of immunization partners at the national, regional and global levels, to adopt relevant recommendations for their settings, and to implement related multicomponent interventions through a health systems approach.

**Table 4:** Recommendations of the SAGE data working group by level and time horizon.^1^

**Recommendation area**	**Specific recommendation**	**Countries**	**Regions**	**Global**	**Time horizon**^1^
1. Embed monitoring of data quality and use into global, regional and national monitoring of immunization and vaccine-preventable disease (VPD) surveillance	WHO to develop a common definition, attributes, and indicators of data quality (i.e., small panel of indicators corresponding to the different data quality attributes) and data use, using those identified in this report as a starting point			x	+
	Integrate ongoing monitoring of data quality and use indicators alongside other routine programme performance (e.g., coverage) and impact indicators	x	x	x	+/++
	Develop and utilize data quality assessment approaches for immunization programme data other than coverage (i.e., VPD surveillance, stock data, etc.)	x	x	x	++
	Evaluate the impact, cost and sustainability of interventions which aim to improve data quality, management, and use to inform decisions on scale-up	x	x	x	++/+++
2. Increase workforce capacity and capability for data quality & use starting at lowest level, where data collection occurs	Develop and disseminate data-related competencies guidance and capacity building tools to implement assessment of workforce at country-level	x	x	x	++/+++
	Ensure data functions (collection, analysis, and use) are accounted for & resourced in workforce management plans, e.g., devoting adequate person-time equivalents, staff recruitment, and retention	x			+++
	Build data capabilities across various levels and career stages (pre-service, refresher, supportive supervision, etc.), considering new approaches (e.g., e-Learning) potential efficiencies created by coordination across programmes	x	x	x	+++
3. Take actions to improve the accuracy of immunization programme targets (denominators)	WHO and UNICEF to revise and finalize the draft guidance on *Assessing and Improving the Accuracy of Target Population Estimates for Immunization Coverage* (2015), including proposing practical and evidence-based solutions			x	++
	Increase immunization programme coordination with national statistics office, birth/civil registration offices, and other relevant programmes/ organizations for improving the quality of denominators	x			++/+++
	Identify and attempt to address the technical (e.g., resident vs non-resident) and non-technical barriers (e.g., political) to accurate denominators in countries, including the use of operational denominators	x	x	x	+++
	Document best practices & country experiences about using different sources (birth cohorts, vital registries & census estimates) or methods for improving denominators	x	x	x	++
4. Enhance use of existing data for tailored action, including immunization programme planning, management and policy-change	At all levels, increase the use of data sources beyond administrative coverage for monitoring, planning and decision-making (e.g., numerators, denominators, surveys, surveillance, vaccine supply, service delivery, serosurveys)	x	x	x	+/++
	Develop /incorporate guidance and training on data triangulation for immunization and surveillance programmes at the national and subnational level	x	x	x	+/++
	Support the development and use of decision-support tools (e.g., monitoring charts, dashboards), as needed, for better planning and programme management	x	x	x	+/++
	Further work on defining the role of serosurveys for immunization programme management at different levels, across different diseases and different epidemiological contexts			x	++
5. Adopt a data-driven continuous quality improvement (CQI) approach as part of health system strengthening	Shift from identifying data quality issues to root cause analysis and improvement planning, as outlined in the draft *Handbook*	x	x	x	++
	Monitor the implementation and impact of previous recommendations to improve accountability and inform new recommendations (e.g. create data-driven improvement cycles)	x	x	x	+/++
	Tailor multi-component strategies for strengthening data collection & use, which may include capacity-building activities, tools, supportive supervision, actionable feedback, staff recognition (e.g. certificates, awards) & accountability mechanisms	x	x	x	++
	Recognize that perverse incentives may have led to overestimation in reported coverage, and ensure that data quality improvements leading to lower coverage are not penalized (i.e., promote accurate reporting)	x	x	x	+++
	Develop a vision and strategic framework for a CQI approach for EPI, including measuring relative changes alongside absolute indicator targets	x	x	x	++/+++
6. Strengthen governance around piloting & implementation of new information, communication, & technology (ICT) tools for immunization & surveillance data collection & use	Design systems and tools based on needs, user requirements, and local context (e.g., sustainability)	x	x	x	+++
	Review existing evidence on cost, impact and effectiveness when considering pilot or scale up new tools for data collection/ management	x	x	x	++
	Plan for and ensure integration & interoperability of any newly introduced tools within the existing information system	x	x	x	+++
	Ensure new information systems include historical data, support all data management functions (archiving, security, and linkage of relevant data), and are accompanied by guidance, standards and specification	x	x	x	+++
7. Improve data sharing and knowledge management across areas and organizations for improved transparency and efficiency	Include best practices on data management (archiving, migration, sharing, and security) in immunization monitoring and surveillance guidance and training	x	x	x	++
	Make data, guidelines, documentation, and reports readily available and accessible to relevant users by building and maintaining user-friendly websites, mobile apps and other communication tools	x	x	x	++
	Improve routine coordination between stakeholders (epidemiologic surveillance, laboratory, and immunization units; private providers, civil society organizations, and partners) with regards to reporting/sharing of relevant data and information	x	x	x	+++
8. WHO & UNICEF to continue strengthening global reporting and monitoring of immunization and surveillance data through a periodic needs assessment and revision process	Continue development and implementation of global (WHO Immunization Information System-WIISE) and regional information systems, including electronic JRF		x	x	+Ongoing
	Collect and monitor disaggregated coverage (e.g., subnational) and surveillance data (e.g., by age, vaccination, lab confirmation)	x	x	x	+Ongoing
	Develop approaches for data collection & routine monitoring of emerging immunization issues, e.g., coverage equity, life-course, migrants / mobile populations, qualitative data		(x)	x	++
	Collaborate to convene new research & validate existing research for improving denominators & national/ subnational coverage (e.g., spatial modelling), including use of data sources beyond coverage (e.g., stock), to inform guidance for programme use			x	++
9. WHO & SAGE should periodically review the implementation status of the WG recommendations, lessons learned, and the gaps to be addressed.				x	Every 2–3 yrs

Abbreviations: EPI = Expanded Programme on Immunization, JRF = WHO/UNICEF Joint Reporting Form on Immunization, SAGE = Strategic Advisory Group of Experts on Immunization, WHO = World Health Organization, WIISE = WHO Immunization Information System, CQI = Continuous Quality Improvement, VPD = Vaccine Preventable Disease, WG=(SAGE Data) Working Group.

1 Time horizon represents a proxy for priority and feasibility. Code is: + short term or within two years; ++ medium term or 2–5 years; +++ long term or 5 or more years.

## 10 Disclaimers

Heather M. Scobie works with the U.S. Centers for Disease Control and Prevention. Use of trade names is for identification only and does not imply endorsement by the Public Health Service or by the U.S. Department of Health and Human Services. The findings and conclusions in this report are those of the authors and do not necessarily represent the official position of the U.S. Centers for Disease Control and Prevention.

M. Carolina Danovaro[-Holliday] works for the World Health Organization. The author alone is responsible for the views expressed in this publication and they do not necessarily represent the decisions, policy or views of the World Health Organization.

All authors declared that they have no known competing financial interests or personal relationships that could have appeared to influence the work reported in this paper. Interests declared by members of the WG were reviewed and conditionally approved by SAGE and the WHO Secretariat [12].

## Declaration of Competing Interest

The authors declare that they have no known competing financial interests or personal relationships that could have appeared to influence the work reported in this paper.

## References

[1] Children's vaccine initiative. Report of the meeting of the scientific advisory group of experts (SAGE), Geneva, 9-11 June 1998, http://apps.who.int/iris/bitstream/handle/10665/64775/WHO_GPV_98.06.pdf?sequence=1; 1988 [Accessed March 9, 2020].

[2] World Health Organization. Meeting of the strategic advisory group of experts on immunization, November 2011 - conclusions and recommendations, https://www.who.int/wer/2012/wer8701.pdf; 2012; 87 (1): 1-16 [Accessed March 9, 2020]. 22242233

[3] Burton A., Monasch R., Lautenbach B., Gacic-Dobo M., Neill M., Karimov R. (2009). WHO and UNICEF estimates of national infant immunization coverage: methods and processes. Bull World Health Organ.

[4] The Strategice Advisory Group of Experts (SAGE) on immunization. SAGE global vaccine action plan assessment reports, https://www.who.int/immunization/global_vaccine_action_plan/sage_assessment_reports/en/; [Accessed April 30, 2020].

[5] World Health Organization. Global routine immunization strategies and practices (GRISP), https://www.who.int/immunization/programmes_systems/policies_strategies/GRISP/en/; 2016 [Accessed April 14, 2020].

[6] World Health Organization. Immunization Agenda 2030: A global strategy to leave no one behind, https://www.who.int/immunization/immunization_agenda_2030/en/; 2020 [Accessed 7 March 200].

[7] Chopra M., Bhutta Z., Chang Blanc D., Checchi F., Gupta A., Lemango E.T., Levine O.S., Lyimo D., Nandy R., O'Brien K.L., Okwo-Bele J.-M., Rees H., Soepardi J., Tolhurst R., Victora C.G. (2020). Addressing the persistent inequities in immunization coverage. Bull World Health Organ.

[8] World Health Organization. Sustainable development goals (SDGs), https://www.who.int/sdg/en/; [Accessed 7 March 2020].

[9] World Health Organization. Univeral health coverage (UHC), https://www.who.int/health-topics/universal-health-coverage#tab=tab_1; [Accessed 7 March 2020].

[10] World Health Organization. Primary health care, https://www.who.int/health-topics/primary-health-care#tab=tab_1; [Accessed April 15, 2020].

[11] World Health Organization. Global framework to strengthen immunization and surveillance data for decision-making (final draft June 2018). 2018.

[12] World Health Organization. SAGE Working group on quality and use of global immunization and surveillance data (established August 2017), https://www.who.int/immunization/policy/sage/sage_wg_quality_use_global_imm_data/en/; 2018 [Accessed December 19, 2018].

[13] World Health Organization (2019). Meeting of the Strategic Advisory Group of Experts on Immunization, October 2019: conclusions and recommendations. Wkly Epidemiol Rec.

[14] Atun RA, Bennett S, Duran A. When do vertical (stand-alone) programmes have a place in health systems? Policy brief, https://apps.who.int/iris/handle/10665/107977; 2008 [Accessed 3 March 2020].

[15] Moher D., Stewart L., Shekelle P. (2015). All in the Family: systematic reviews, rapid reviews, scoping reviews, realist reviews, and more. Syst Rev.

[16] Report of the SAGE working group on quality and use of immunization and surveillance data (Sept. 2019), https://www.who.int/immunization/sage/meetings/2019/october/presentations_background_docs/en/; [Accessed April 30, 2020].

[17] Bloland P, MacNeil A. Defining & assessing the quality, usability, and utilization of immunization data BMC Public Health. 2019;19:380. 10.1186/s12889-019-6709-1 PMC6450010 30947703

[18] World Health Organization. Vaccination coverage cluster surveys: reference manual, https://apps.who.int/iris/handle/10665/272820; 2018 [Accessed March 8, 2020].

[19] World Health Organization. Inequality monitoring in immunization: a step-by-step manual, https://www.who.int/gho/health_equity/manual_immunization/en/; 2019 [Accessed April 29, 2020].

[20] Bosch-Capblanch X., Ronveaux O., Doyle V., Remedios V., Bchir A. (2009). Accuracy and quality of immunization information systems in forty-one low income countries. Trop Med Int Health.

[21] Dolan S.B., MacNeil A. (2017). Comparison of inflation of third dose diphtheria tetanus pertussis (DTP3) administrative coverage to other vaccine antigens. Vaccine.

[22] Lim S.S., Stein D.B., Charrow A., Murray C.JL. (2008). Tracking progress towards universal childhood immunisation and the impact of global initiatives: a systematic analysis of three-dose diphtheria, tetanus, and pertussis immunisation coverage. The Lancet.

[23] MacNeil A., Dietz V., Cherian T. (2014). Vaccine preventable diseases: Time to re-examine global surveillance data?. Vaccine.

[24] Murray C.JL., Shengelia B., Gupta N., Moussavi S., Tandon A., Thieren M. (2003). Validity of reported vaccination coverage in 45 countries. The Lancet.

[25] Ronveaux O., Rickert D., Hadler S., Groom H., Lloyd J., Bchir A. (2005). The immunization data quality audit: verifying the quality and consistency of immunization monitoring systems. Bull World Health Organ.

[26] Clarke K.E.N., MacNeil A., Hadler S., Scott C., Tiwari T.S.P., Cherian T. (2019). Global epidemiology of Diphtheria, 2000–2017. Emerg Infect Dis.

[27] Brown DW. A comparison of national immunization programme target population estimates with data from an independent source and differences in computed coverage levels for the third dose of DTP containing vaccine. World J Vaccines 2014;4:18–23.

[28] Stashko LA, Gacic-Dobo M, Dumolard LB, Danovaro-Holliday MC. Assessing the quality and accuracy of national immunization program reported target population estimates from 2000 to 2016. PLoS One 2019;14:e0216933 10.1371/journal.pone.0216933 PMC6615593 31287824

[29] Phalkey R.K., Yamamoto S., Awate P., Marx M. (2015). Challenges with the implementation of an Integrated disease surveillance and response (IDSR) system: systematic review of the lessons learned. Health Policy and Planning.

[30] Wilkins K, Nsubuga P, Mendlein J, Mercer D, Pappaioanou M. The data for decision making project: assessment of surveillance systems in developing countries to improve access to public health information. Public Health 2008;122:914–22 10.1016/j.puhe.2007.11.002 18490035

[31] World Health Organization. Landscape Assessment of the immunization information systems for the World Health Organization (WHO) Headquarters (HQ) and Regional Offices. 2017

[32] World Health Organization. Subnational immunization coverage data, https://www.who.int/immunization/monitoring_surveillance/data/subnational/en/; [Accessed March 7, 2019].

[33] World Health Organization. Surveillance Standards for vaccine preventable diseases http://www.who.int/immunization/monitoring_surveillance/burden/vpd/standards/en/; 2018 [Accessed April 30, 2020].

[34] World Health Organization. WHO/UNICEF joint reporting process, https://www.who.int/immunization/monitoring_surveillance/routine/reporting/en/; [Accessed March 7, 2020].

[35] World Health Organization (M. Gacic-Dobo). Mapping of global immunization programme data: gaps and opportunities (presentation to SAGE, Oct. 2018), http://origin.who.int/immunization/sage/meetings/2018/october/SAGE_october_2018_diphtheria_GacicDobo.pdf; [Accessed March 7, 2020].

[36] World Health Organization. WHO/UNICEF estimates of national immunization coverage, https://www.who.int/immunization/monitoring_surveillance/routine/coverage/en/index4.html; [Accessed March 7, 2020].

[37] UNICEF. Immunization, https://data.unicef.org/topic/child-health/immunization/; [Accessed March 7, 2020].

[38] World Health Organization. Immunization, vaccines and biologicals: monitoing and surveillance, https://www.who.int/immunization/monitoring_surveillance/en/; [Accessed March 7, 2020].

[39] TechNet-21. WHO EPI - core reference material, https://www.technet-21.org/en/topics/epi-core-reference; 2019 [Accessed April 29, 2020].

[40] World Health Organization (WHO). Data quality review, http://www.who.int/healthinfo/tools_data_analysis/dqr_modules/en/; 2017 [Accessed September 18, 2018].

[41] World Health Organization (WHO). The immunization data quality self-assessment (DQS) tool, http://www.who.int/immunization/monitoring_surveillance/routine/coverage/DQS_tool.pdf; 2005 [Accessed March 9, 2020].

[42] Immunization Data Evidence for Action (IDEA). A realist review of what works to improve data use for immunization, evidence from low- and middle-income countries, https://www.path.org/resources/immunization-data-evidence-action-realist-review-what-works-improve-data-use-immunization/; 2019 [Accessed April 30, 2020].

[43] Wetherill O, Lee C, Dietz V. Root causes of poor immunisation data quality and proven interventions: a systematic literature review. Ann Infect Dis Epidemiol 2017;2 PMC10719814 38098515

[44] Harrison K, Rahimi N, Danovaro-Holliday MC. Factors limiting data quality in the expanded programme on immunization in low and middle-income countries: a scoping review. Vaccine 2020;38:4652–63 10.1016/j.vaccine.2020.02.091 32446834

[45] MEASURE Evaluation. Barriers to use of health data in low- and middle- income countries a review of the literature (working paper), https://www.measureevaluation.org/resources/publications/wp-18-211; 2018 [Accessed April 29, 2020].

[46] Edelstein M., Lee L.M., Herten-Crabb A., Heymann D.L., Harper D.R. (2018). Strengthening global public health surveillance through data and benefit sharing. Emerg Infect Dis.

[47] van Panhuis W.G., Paul P., Emerson C., Grefenstette J., Wilder R., Herbst A.J., Heymann D., Burke D.S. (2014). A systematic review of barriers to data sharing in public health. BMC Public Health.

[48] Arenth B, Bennett A, Bernadotte C, Carnahan E, Dube M, Thompson J, et al. Defining and building a data use culture, http://bidinitiative.org/wp-content/uploads/PATH_Building-Data-Use-Culture_R1.pdf; 2017 [Accessed March 9, 2020].

[49] Brown D.W., Burton A.H., Feeny G., Gacic-Dobo M. (2014). Avoiding the will o’ the wisp: challenges in measuring high levels of immunization coverage with precision. World J Vaccines.

[50] Mitrovich R, Marti M, Watkins M, Duclos P. A review of the private sector’s contribution to immunization service delivery in low, middle, and high-income countries (presentation to SAGE, April 2017), https://www.who.int/immunization/sage/meetings/2017/april/2_Review_private_sector_engagement_Mitrovich_et_al.pdf?ua; 2017 [Accessed March 7, 2020].

[51] World Health Organization. Universal health coverage across the life course, https://www.who.int/life-course/en/; [Accessed April 14, 2020].

[52] Equity Reference Group for Immunization (ERG). Equity reference group for immunization, https://sites.google.com/view/erg4immunisation/home; [Accessed April 29, 2020].

[53] Pan American Health Organization (PAHO). Electronic immunization registry: practical considerations for planning, development, implementation and evaluation, http://iris.paho.org/xmlui/handle/123456789/34865; 2018 [Accessed March 9, 2019].

[54] World Health Organization. Handbook on the use, collection, and improvement of immunization data (2019 working draft); 2018.

[55] The Geneva Learning Foundation. WHO immunization monitoring academy information, https://learning.foundation/ima-level1-en/?utm_source=WHO+Scholar+network+%28English%29&utm_campaign=e9e7e0da24-EMAIL_CAMPAIGN_2018_05_28_05_22_COPY_01&utm_medium=email&utm_term=0_55bba48b4a-e9e7e0da24-260659585; 2018 [Accessed April 14, 2020].

[56] World Health Organization. Immunization training resources, https://www.who.int/immunization/documents/training/en/; [Accessed April 14, 2020].

[57] World Health Organization. Global strategy on comprehensive vaccine-preventable disease surveillance (2020 draft), Available upon request vpdata@who.int; [Accessed

[58] Danovaro-Holliday M.C., Ortiz C., Cochi S., Ruiz-Matus C. (2014). Electronic immunization registries in Latin America: progress and lessons learned. Rev Panam Salud Publica.

[59] European Centre for Disease Prevention and Control (ECDC). Information systems to record information about vaccination, https://www.ecdc.europa.eu/en/immunisation-vaccines/immunisation-information-systems; [Accessed April 14, 2020].

[60] TechNet-21. Introducing digital immunization information systems–exchange and learning from Vietnam (IDEAL-Vietnam) project, https://www.technet-21.org/en/topics/ideal; [Accessed April 30, 2020].

[61] Gilbert S.S., Bulula N., Yohana E., Thompson J., Beylerian E., Werner L., Shearer J.C. (2020). The impact of an integrated electronic immunization registry and logistics management information system (EIR-eLMIS) on vaccine availability in three regions in Tanzania: A pre-post and time-series analysis. Vaccine.

[62] Ahmed D. Use of data to guide India's immunization programme (presentation to SAGE, Oct. 2019), https://www.who.int/immunization/sage/meetings/2019/october/ahmed_data_sage_october_2019.pdf?ua=1; [Accessed April 14, 2020].

[63] Institute. CUE. GRID3 project aims to put everyone on the map, https://blogs.ei.columbia.edu/2019/12/04/grid3-population-mapping/; 2019 [Accessed March 8, 2020].

[64] Wardrop N.A., Jochem W.C., Bird T.J., Chamberlain H.R., Clarke D., Kerr D., Bengtsson L., Juran S., Seaman V., Tatem A.J. (2018). Spatially disaggregated population estimates in the absence of national population and housing census data. Proc Natl Acad Sci USA.

[65] Maina I., Wanjala P., Soti D., Kipruto H., Droti B., Boerma T. (2017). Using health-facility data to assess subnational coverage of maternal and child health indicators, Kenya. Bull World Health Organ.

[66] Maternal and Child Survival Program (MCSP). Mozambique program brief: addressing the denominator conundrum for maternal and child health programs: a New Methodology, https://www.mcsprogram.org/wp-content/uploads/dlm_uploads/2019/01/MCSP-MZ-Brief-TargetPopulationMethodology.pdf; [Accessed April 15, 2020].

[67] Ghiselli M.E., Wilson I.N., Kaplan B., Waziri N.E., Sule A., Ayanleke H.B. (2019). Comparison of micro-census results for magarya ward, wurno local government area of Sokoto State, Nigeria, with Other Sources of Denominator Data. Data (Basel).

[68] Correa G., Verstraete P., Soundardjee R., Shankar M., Paterson C., Hampton L. (2019). Immunization programmes and notifications of vital events. Bull World Health Organ.

[69] World Health Organization. Civil registration and vital statistics (CRVS) Resource Kit, https://www.who.int/healthinfo/civil_registration/en/; 2012 [Accessed 15 January 2019].

[70] Rahman M.H., Cox A.B., Mills S.L. (2019). A missed opportunity: birth registration coverage is lagging behind Bacillus Calmette-Guerin (BCG) immunization coverage and maternal health services utilization in low- and lower middle-income countries. J Health Popul Nutr.

[71] Pan American Health Organization (PAHO). PAHO immunization newsletter (December 2012), https://www.paho.org/hq/dmdocuments/2013/SNE3406.pdf; 2012 [Accessed April 30, 2020].

[72] Mikkelsen L., Phillips D.E., AbouZahr C., Setel P.W., de Savigny D., Lozano R., Lopez A.D. (2015). A global assessment of civil registration and vital statistics systems: monitoring data quality and progress. The Lancet.

[73] Rowe A.K., Rowe S.Y., Peters D.H., Holloway K.A., Chalker J., Ross-Degnan D. (2018). Effectiveness of strategies to improve health-care provider practices in low-income and middle-income countries: a systematic review. The Lancet Global Health.

[74] World Health Organization. Health system governance, https://www.who.int/healthsystems/topics/stewardship/en/ [Accessed February 19, 2019].

[75] World Health Organization (WHO). WHO accountability framework (March 2015), https://www.who.int/about/finances-accountability/accountability/accountability-framework.pdf; [Accessed April 14, 2020].

[76] Dumit E.M., Novillo-Ortiz D., Contreras M., Velandia M., Danovaro-Holliday M.C. (2018). The use of eHealth with immunizations: An overview of systematic reviews. Vaccine.

[77] Trumbo S.P., Contreras M., García A.G.F., Díaz F.A.E., Gómez M., Carrión V., Ruiz K.J.P., Aquije R., Danovaro-Holliday M.C., Velandia-González M. (2018). Improving immunization data quality in Peru and Mexico: Two case studies highlighting challenges and lessons learned. Vaccine.

[78] World Health Organization. Global framework for immunization monitoring and surveillance (GFIMS) https://apps.who.int/iris/handle/10665/69685; 2007 [Accessed June 26, 2020]. 10.2471/BLT.07.048223 PMC2636314 18278243

[79] Cutts F.T., Izurieta H.S., Rhoda D.A. (2013). Measuring coverage in MNCH: design, implementation, and interpretation challenges associated with tracking vaccination coverage using household surveys. PLoS Med.

[80] Kak N, Burkhalter B, Cooper M. Measuring the competence of healthcare providers. In: Project QAQ, editor. Operations Research Issue Paper 2(1). Bethesda, MD: USAID; 2001

[81] Nicol E, Turawa E, Bonsu G. Pre- and in-service training of health care workers on immunization data management in LMICs: a scoping review. Hum Resour Health. 2019;17:92 10.1186/s12960-019-0437-6 PMC6889656 31791352

[82] Loignon C., Hudon C., Goulet É., Boyer S., De Laat M., Fournier N., Grabovschi C., Bush P. (2015). Perceived barriers to healthcare for persons living in poverty in Quebec, Canada: the EQUIhealThY project. Int J Equity Health.

[83] Nicol E., Bradshaw D., Phillips T., Dudley L. (2013). Human factors affecting the quality of routinely collected data in South Africa. Stud Health Technol Inform.

[84] Ward K., Mugenyi K., Benke A., Luzze H., Kyozira C., Immaculate A. (2017). Enhancing workforce capacity to improve vaccination data quality, Uganda. Emerg Infect Dis.

[85] Whittaker M., Hodge N., Mares R.E., Rodney A. (2015). Preparing for the data revolution: identifying minimum health information competencies among the health workforce. Hum Resour Health.

[86] Ledikwe J.H., Grignon J., Lebelonyane R., Ludick S., Matshediso E., Sento B.W., Sharma A., Semo B.-W. (2014). Improving the quality of health information: a qualitative assessment of data management and reporting systems in Botswana. Health Res Policy Sys.

[87] Uwimana J, Zarowsky C, Hausler H, Jackson D. Training community care workers to provide comprehensive TB/HIV/PMTCT integrated care in KwaZulu-Natal: lessons learnt. Trop Med Int Health. 2012;17:488-96 10.1111/j.1365-3156.2011.02951.x 22296235

[88] St Louis M. Global health surveillance. MMWR Suppl. 2012;61:15-9 22832992

[89] English R., Masilelai T., Barron P., Schönfeldt A., Padarath A., English R. (2011). Health information systems in South Africa. South African health review 2011.

[90] Avortri GS, Nabukalu JB, Nabyonga-Orem J. Supportive supervision to improve service delivery in low-income countries: is there a conceptual problem or a strategy problem? BMJ Glob Health 2019;4:e001151 10.1136/bmjgh-2018-001151 PMC6797347 31673434

[91] Vasan A., Mabey D.C., Chaudhri S., Brown Epstein H.A., Lawn S.D. (2017). Support and performance improvement for primary health care workers in low- and middle-income countries: a scoping review of intervention design and methods. Health Policy Planning.

[92] Drehobl P.A., Roush S.W., Stover B.H., Koo D. (2012). Centers for Disease C, Prevention. Public health surveillance workforce of the future. MMWR Suppl.

[93] Traicoff D., Pope A., Bloland P., Lal D., Bahl J., Stewart S. (2019). Developing standardized competencies to strengthen immunization systems and workforce. Vaccine.

[94] World Health Organization. Immunization competencies initiative: competencies of the immunization technical workforce - draft for SAGE meeting April 2017 https://www.who.int/immunization/sage/meetings/2017/april/1_CDC_GID_Immunization_CompetenciesSAGE_mtg.pdf; 2017 [Accessed March 9, 2019].

[95] Ledikwe J.H., Reason L.L., Burnett S.M., Busang L., Bodika S., Lebelonyane R. (2013). Establishing a health information workforce: innovation for low- and middle-income countries. Hum Resour Health.

[96] World Health Organization Regional Office for Africa. Expanded programme on immunization prototype curriculum for medical schools in the WHO African Region (Update December 2015), http://www.who.int/iris/handle/10665/250674; 2015 [Accessed April 30, 2020].

[97] World Health Organization Regional Office for Africa. Expanded programme on immunization prototype curriculum for nursing/widwifery schools in the WHO African Region (Update December 2015), https://apps.who.int/iris/handle/10665/250671; 2015 [Accessed April 30, 2020].

[98] World Health Organization regional office for Africa. Mid-level management course for EPI managers, https://www.afro.who.int/publications/mid-level-management-course-epi-managers; 2017 [Accessed February 16, 2019].

[99] Borgermans ML. Strengthening a competent health workforce for the provision of coordinated/integrated health services, http://www.euro.who.int/__data/assets/pdf_file/0010/288253/HWF-Competencies-Paper-160915-final.pdf; 2005 [Accessed April 30, 2020].

[100] District Health Information Systems 2 (DHIS2). DHIS2 in Action, https://www.dhis2.org/inaction; [Accessed March 3, 2020].

[101] World Health Organization/PATH. Planning an information systems project, https://www.who.int/immunization/programmes_systems/supply_chain/optimize/planning_information_systems_project.pdf; 2013 [Accessed February 19, 2019].

[102] Namageyo-Funa A., Samuel A., Bloland P., Macneil A. (2018). Considerations for the development and implementation of electronic immunization registries in Africa. Pan Afr Med J.

[103] Poy A, van den Ent M, Sosler S, Hinman AR, Brown S, Sodha S, et al. Monitoring results in routine immunization: development of routine immunization dashboard in selected african countries in the context of the polio eradication endgame strategic plan. J Infect Dis 2017;216:S226-S36 10.1093/infdis/jiw635 PMC5853840 28838180

[104] World Health Organization (WHO). Guideline recommendations on digital interventions for health system strengthening, https://apps.who.int/iris/bitstream/handle/10665/311941/9789241550505-eng.pdf?ua=1; 2019 [Accessed April 15, 2020]. 31162915

[105] World Health Organization, UNICEF, centers for disease control and prevention. Public health data triangulation for immunization and vaccine-preventable disease surveillance programs: framework document (Dec. 2019 working document), https://www.learning.foundation/vpd-triangulation-draft; [Accessed March 7, 2020].

[106] Edelstein M., White J., Bukasa A., Saliba V., Ramsay M. (2019). Triangulation of measles vaccination data in the United Kingdom of Great Britain and Northern Ireland. Bull. World Health Organ.

[107] The Geneva Learning Foundation. WHO Scholar Level 2 certification course on data triangulation for improved decision making in immunization programmes, https://old.learning.foundation/who-data-improvement-level-2-en/; 2020 [Accessed].

[108] Wagenaar B.H., Hirschhorn L.R., Henley C., Gremu A., Sindano N., Chilengi R. (2017). Data-driven quality improvement in low-and middle-income country health systems: lessons from seven years of implementation experience across Mozambique, Rwanda, and Zambia. BMC Health Serv Res.

[109] Schwartz S.P., Rehder K.J. (2017). Quality improvement in pediatrics: past, present, and future. Pediatr Res.

[110] Gilkey M.B., Moss J.L., Roberts A.J., Dayton A.M., Grimshaw A.H., Brewer N.T. (2014). Comparing in-person and webinar delivery of an immunization quality improvement program: a process evaluation of the adolescent AFIX trial. Implement Sci.

[111] U.S. Centers for Disease Control and Prevention. (IQIP) immunization quality improvement for providers, https://www.cdc.gov/vaccines/programs/iqip/index.html; [Accessed 3 March 2020].

[112] BID Initiative. Webinar: RED-QI (Reaching Every District incorporating Quality Improvement), https://bidinitiative.org/events/event/webinar-red-qi-reaching-every-district-incorporating-quality-improvement/; 2014 [Accessed].

[113] John Snow Inc (JSI). Reaching every district using quality improvement methods (RED-QI): A guide for immunization programme managers (May 2015), https://mpffs6apl64314hd71fbb11y-wpengine.netdna-ssl.com/wp-content/uploads/2015/05/UI-FHS_HowtoGuide.pdf; [Accessed April 30, 2020].

[114] UNICEF. DIVA Guidebook: Strengthening district management for results with equity, https://www.childhealthtaskforce.org/resources/report/2012/diva-guidebook-strengthening-district-management-results-equity-unicefmsh; 2012 [Accessed April 30, 2020].

[115] Manyazewal T., Mekonnen A., Demelew T., Mengestu S., Abdu Y., Mammo D. (2018). Improving immunization capacity in Ethiopia through continuous quality improvement interventions: a prospective quasi-experimental study. Infect Dis Poverty.

[116] Alfonso V.H., Bratcher A., Ashbaugh H., Doshi R., Gadoth A., Hoff N. (2019). Changes in childhood vaccination coverage over time in the Democratic Republic of the Congo. PLoS ONE.

[117] Dunkle S.E., Wallace A.S., MacNeil A., Mustafa M., Gasasira A., Ali D., Elmousaad H., Mahoney F., Sandhu H.S. (2014). Limitations of using administratively reported immunization data for monitoring routine immunization system performance in Nigeria. J Infect Dis.

[118] World Health Organization African Regional Office. Business case for WHO immunization activities on the African continent 2018-2030, https://www.afro.who.int/publications/business-case-who-immunization-activities-african-continent-2018-2030; 2018 [Accessed March 4, 2019].

[119] World Health Organization Regional Office for Africa. Investment case for vaccine-preventable diseases surveillance in the African Region 2020-2030, https://www.afro.who.int/publications/investment-case-vaccine-preventable-diseases-surveillance-african-region-2020-2030; 2019 [Accessed March 9, 2020].

[120] Lange C., Range B., Welsh K. (2012). Conditions for effective data use to improve schools: recommendations for school leaders. Int J Educ Leadership Preparation.

[121] Wayman JC, Brewer C, Stringfield S. Leadership for effective data use, http://www.waymandatause.com/wp-content/uploads/2013/11/Wayman_Brewer_Stringfield_AERA2009.pdf; 2009 [Accessed March 9, 2020].

[122] BID Initiative. BID Initiative: Better data, better decisions, better health, https://bidinitiative.org/; [Accessed April 30, 2020].

[123] MEASURE evaluation. PRISM: Performance of routine information system management, https://www.measureevaluation.org/resources/tools/health-information-systems/prism; 2019 [Accessed April 29, 2020].

[124] World Health Organization (WHO). The immunization data quality audit (DQA) procedure, http://apps.who.int/iris/bitstream/handle/10665/68462/WHO_V-B_03.19_eng.pdf?sequence=1; 2003 [Accessed March 9, 2020].

[125] World Health Organization (WHO). Assessing and improving the accuracy of target population estimates for immunization coverage (working draft), http://www.who.int/immunization/monitoring_surveillance/data/Denominator_guide.pdf?ua=1; 2015 [Accessed March 9, 2020].

[126] World Health Organization (WHO). Guide to the health facility data quality report card, http://www.who.int/healthinfo/DQRC_Indicators.pdf; 2015 [Accessed March 9, 2020].

[127] Pan American Health Organization (PAHO). Tools for monitoring the coverage of integrated public health interventions: Vaccination and deworming of soil-transmitted helminthiasis. , http://iris.paho.org/xmlui/handle/123456789/34510; 2017 [Accessed March 9, 2020].

[128] World Health Organization. Analysis and use of health facility data: guidance for immunization programme managers (working document, February 2018), https://www.who.int/healthinfo/tools_data_analysis_routine_facility/en/; 2018 [Accessed March 9, 2020].

[129] Danovaro-Holliday M.C., Dansereau E., Rhoda D.A., Brown D.W., Cutts F.T., Gacic-Dobo M. (2018). Collecting and using reliable vaccination coverage survey estimates: Summary and recommendations from the “Meeting to share lessons learnt from the roll-out of the updated WHO Vaccination Coverage Cluster Survey Reference Manual and to set an operational research agenda around vaccination coverage surveys”, Geneva, 18–21 April 2017. Vaccine.

